# Infective endocarditis in the Netherlands: current epidemiological profile and mortality: An analysis based on partial ESC EORP collected data

**DOI:** 10.1007/s12471-020-01431-z

**Published:** 2020-06-05

**Authors:** S. El Kadi, D. M. F. van den Buijs, T. Meijers, M. D. Gilbers, S. C. A. M. Bekkers, J. P. van Melle, R. K. Riezebos, W. L. Blok, W. Tanis, A. R. Wahadat, J. W. Roos-Hesselink, T. I. G. van der Spoel, S. A. J. Chamuleau, O. Kamp, EURO ENDO Investigators group

**Affiliations:** 1 location VU University Medical Centre, Department of Cardiology, Amsterdam UMC, Amsterdam University Medical Centers, Amsterdam, The Netherlands; 2 Department of Cardiothoracic surgery, Maastricht UMC, Maastricht University Medical Center+, Maastricht, The Netherlands; 3 Department of Cardiology, Maastricht UMC, Maastricht University Medical Center+, Maastricht, The Netherlands; 4 Department of Cardiology, UMCG, University Medical Centre Groningen, Groningen, The Netherlands; 5 Department of Cardiology, OLVG, Onze Lieve Vrouwe Gasthuis—Location Oost, Amsterdam, The Netherlands; 6 Department of Internal Medicine, OLVG, Onze Lieve Vrouwe Gasthuis—Location Oost, Amsterdam, The Netherlands; 7 Department of Cardiology, Haga Teaching Hospital, The Hague, The Netherlands; 8 Department of Cardiology, Erasmus MC, University Medical Centre Rotterdam, Rotterdam, The Netherlands; 9 Department of Cardiology, UMC Utrecht, University Medical Centre Utrecht, Utrecht, The Netherlands

**Keywords:** Infective endocarditis, Dutch registry, Prosthetic valve endocarditis, Imaging, Mortality

## Abstract

**Introduction:**

Infective endocarditis (IE) is associated with a high in-hospital and long term mortality. Although progress has been made in diagnostic approach and management of IE, morbidity and mortality of IE remain high. In the latest European guidelines, the importance of the multi-modality imaging in diagnosis and follow up of IE is emphasized.

**Aim:**

The aim was to provide information regarding mortality and adverse events of IE, to determine IE characteristics and to assess current use of imaging in the diagnostic workup of IE.

**Methods:**

This is a prospective observational cohort study. We used data from the EURO-ENDO registry. Seven hospitals in the Netherlands have participated and included patients with IE between April 2016 and April 2018.

**Results:**

A total of 139 IE patients were included. Prosthetic valve endocarditis constituted 32.4% of the cases, cardiac device related IE 7.2% and aortic root prosthesis IE 3.6%. In-hospital mortality was 14.4% (20 patients) and one-year mortality was 21.6% (30 patients). The incidence of embolic events under treatment was 16.5%, while congestive heart failure or cardiogenic shock occurred in 15.1% of the patients. Transthoracic and transoesophageal echocardiography were performed most frequently (97.8%; 81.3%) and within 3 days after IE suspicion, followed by ^18^F‑fluorodeoxyglucose positron emission tomography/computed tomography (45.3%) within 6 days and multi-slice computed tomography (42.4%) within 7 days.

**Conclusion:**

We observed a high percentage of prosthetic valve endocarditis, rapid and extensive use of imaging and a relatively low in-hospital and one-year mortality of IE in the Netherlands. Limitations include possible selection bias.

**Electronic supplementary material:**

The online version of this article (10.1007/s12471-020-01431-z) contains supplementary material, which is available to authorized users. A complete list of the EURO-ENDO Investigators Group and of the EURO-ENDO National Coordinators is provided in the ESM.

## What’s new?

- This is a prospective multi-centre observational study on infective endocarditis in the Netherlands
- One-third of the patients have prosthetic valve endocarditis
- Transthoracic and transoesophageal echocardiography are frequently used for the diagnostic workup (97.8% and 81.3% with at least one examination), followed by PET-CT (45.3%)
- Surgery is performed in 50.1% of the patients, more than half of these patients received early surgery (i.e. <14 days)
- In-hospital mortality is 14.4% and 1‑year mortality is 21.6%

## Introduction

Infective endocarditis (IE) is an infectious disease with high mortality and morbidity [1]. The epidemiologic profile of IE has changed over the past decades. While IE previously affected young patients with preexisting (rheumatic) valvular abnormalities [2], current risk factors include exposure to health care associated procedures, old age, prosthetic valves and intracardiac devices [3, 4]. The exact incidence of IE is difficult to determine since definitions of IE have changed over time. In the Netherlands, a retrospective epidemiological study demonstrated an increase in incidence of IE from 2005 till 2011 [5], coinciding with the more restrictive use of antibiotic prophylaxis as recommended by the European Society of Cardiology (ESC) [6]. In the 2015 ESC guidelines a number of preventive, diagnostic and therapeutic adjustments were proposed [7]. Some of the key recommendations were early transthoracic (TTE) and transoesophageal echocardiography (TOE) and early surgery for prevention of heart failure, structural damage and embolic events. More importantly, novel major criteria have been added to the modified Duke criteria. The modified ESC 2015 diagnostic criteria now includes paravalvular lesions identified with cardiac computed tomography (cardiac CT) and abnormal activity around the implantation site of prosthetic valves seen with ^18^F‑fluorodeoxyglucose positron emission tomography/computed tomography (^18^F‑FDG PET/CT). In aortic IE, cardiac CT has the advantage of providing information regarding the extent of IE as well as the anatomy of the aortic structures and coronary arteries before surgery. The use of additional imaging modalities allows for earlier and reliable diagnosis of IE, particularly in the setting of prosthetic valves or when echocardiography is inconclusive [8]. Also, since IE may present with different symptoms depending on the affected organs and complications, a multidisciplinary approach is essential. Early and regular discussion of IE patients in the Endocarditis Team may lead to early additional imaging and correct interpretation, altered antimicrobial strategies and early surgery. Different observational studies suggested that a collaborative approach by an Endocarditis Team results in a lower mortality [9, 10]. Current in-hospital mortality rate is being estimated at approximately 20% [1, 11]. In the Netherlands, there are no recent data regarding in-hospital and one-year mortality. We aim to provide information regarding mortality and adverse events of IE in the Netherlands and assess IE characteristics and current use of imaging modalities in the diagnostic workup of IE. The outcome of this study may contribute to a better insight in present IE profile and clinical practice and provide outcomes on hard clinical endpoints in light of the recent changes in IE management.

## Methods

We conducted a prospective observational multi-center cohort study using data of the European Endocarditis Registry (EURO-ENDO). This registry has been initiated by the ESC with the aim to study the current diagnostic and therapeutic practice of IE across Europe and outside. Patients aged eighteen years and over with definite IE or possible IE (according to the modified ESC 2015 diagnostic criteria) were included in the EURO-ENDO. Inclusion was between April 2016 and April 2018. Follow up lasted until April 2019. In the Netherlands, seven hospitals with a combined adherence region of 8 million inhabitants, participated in the EURO-ENDO registry. Each center included patients in a time frame of one year. Variables that were collected include demographics, relevant pre-existing valve conditions, laboratory and imaging findings, antimicrobial therapy, surgery and follow up. Approval of the medical ethics committee was obtained before data collection in all centers. All participating patients provided informed consent.

The primary endpoint is in-hospital and one-year mortality. Secondary endpoints are incidence of embolic events, congestive heart failure and cardiogenic shock during hospital stay.

Descriptive summaries of the data are presented. Continuous variables are expressed as mean and standard deviation or median and interquartile range. Categorical variables are expressed in percentages. Student’s t‑test or non-parametric tests were used to compare continuous data between groups, Fisher’s exact test to compare categorical data. In addition, we performed multivariable analyses using logistic regression to correctly assess association between the variables and the endpoints. SPSS (v. 22) was used for analysis.

## Results

### Patient characteristics

Between 2016 and 2018, 139 patients from the Netherlands have been included in the EURO-ENDO registry. From these patients, approximately 40% were referred from regional hospitals to one of the participating cardiac surgery hospitals. The clinical characteristics of the patients are presented in Tab. 1. The majority of the patients were men (68.3%) with a median age of 64 years (IQR 57.0–75.0). Of the patients presenting with IE, 9 patients (6.5%) had a previous episode of endocarditis. Approximately one third (33.8%) had previous valve surgery. At presentation, 33.1% of the patients had a prosthetic valve. Three patients (2%) had previous trans-aortic valve implantation. A cardiac device was in place in 17 patients (12.2%). Ten patients (14.1%) had a congenital heart disease. There were 117 patients (83.5%) with definite IE and 22 patients (16.5%) with possible IE.

**Table 1:** Baseline characteristics

Characteristics	*N* = 139 (%)
Male	95 (68.3)
Mean age	63.9 (57.0–75.0)
Previous endocarditis	9 (6.5)
Congenital heart disease	14 (10.1)
Previous valve surgery	47 (33.8)
Prosthetic valve at admission	46 (33.1)
Device therapy at admission	17 (12.2)
Positive culture	127 (91.4)
Positive culture, major criterium	106 (76.3)
Positive culture, minor criterium	21 (15.1)
Staphylococcus aureus	31 (22.3)
Streptococci	59 (42.4)
Enterococci	16 (11.5)
Other	24 (17.3)
Native valve endocarditis	85 (61.2)
Prosthetic valve endocarditis	45 (32.4)
Cardiac device related IE	10 (7.2)
Aortic root/ascending aorta prosthesis IE	5 (3.6)
Aortic valve IE	87 (62.6)
Mitral valve IE	41 (29.5)
Pulmonary valve IE	6 (4.3)
Tricuspid valve IE	3 (2.2)
Surgery	70 (50.4)
Possible IE	23 (16.5)
Definite IE	116 (83.5)

Values expressed as *N* (percentage) and median (interquartile range)

### Microbiology

Of the patients with IE, 91.4% had positive blood cultures. In 76.3% of the cases the blood cultures met the major criterium of the modified Dukes criteria, whereas 15.1% fulfilled the minor criterium. Staphylococcus aureus (SA) was the most frequently cultured micro-organism (31 patients; 22.3%). As a group, the streptococci were most prevalent (42.4%). There were 35 patients with streptococcus viridans (25.2%). Among the other cultured pathogens were enterococci (11.5%), Propionibacterium acnes (*n* = 2) and gram negative bacilli (*n* = 2). Streptococcus related IE was seen more often in NVE compared with PVE (48% vs. 31.7%, *P* = 0.09; Suppl. Table 3). Enterococci and coagulase negative staphylococci were more frequently found in PVE patients (19.5% vs. 8.2%, *P* = 0.08 and 14.6% vs. 2.0%, *P* < 0.01 respectively).

### Affected valves

NVE represented 56.8% of the cases, while 32.4% of the patients had PVE. We also observed 10 cases (7.2%) of cardiac device related IE (CDRIE) and 5 cases (3.6%) of IE of the aortic root or ascending aorta prosthesis. In 87 patients (62.6%), the aortic valves were affected. Mitral valves were affected in 41 patients (29.5%). Pulmonary and tricuspid valves were affected in 4.3% and 2.3% of the patients respectively. Combined aortic and mitral valve IE occurred in 15 patients (10.8%).

### Imaging

Various imaging modalities were used for diagnosing IE (Tab. 2). Transthoracic echocardiography (TTE) was performed at least once in 136 patients (97.8%) and transoesophageal echocardiography (TOE) in 113 patients (81.3%). Overall use of multislice computed tomography (MS-CT) was 42.4%, the use of cardiac MS-CT scan was 25.9%. Thirty-one patients (22.3%) had an MRI-scan to detect embolic complications. Fifteen patients had an MRI scan of the brain and 18 patients had an MRI scan of the spine. One MRI scan of the abdomen was made. Cardiac MRI was not performed. ^18^F‑FDG PET/CT scan was performed significantly more often in patients with prosthetic valves or devices compared to NVE patients (60.0% vs. 30.6%, *P* < 0.001). In total 63 patients (45.3%) received a ^18^F‑FDG PET/CT scan. TTE was usually performed the same day that the diagnosis IE was suspected (0–2). The median time until performing TOE was three days (1–6). Cardiac MS-CT was performed after 7 days (2–11.8), PET-CT scan after 6 days (3–10).

**Table 2:** Use and timing of imaging

Imaging modality	No of patients with at least one exam performed (%)				Median time (days) after date of suspected IE (IQR)
	Total^a^	NVE	PVE	*P*-value	
Transthoracic echocardiography (TTE)	136 (97.8)	84 (98.8%)	44 (97.8%)	>0.999	0 (0–2)
Transoesophageal echocardiography (TOE)	113 (81.3)	65 (76.5%)	37 (82.2%)	0.056	3 (1–6)
Multislice CT-scan (total)	59 (42.4)	32 (37.6%)	25 (55.6%)	0.064	5 (1–11)
Multislice CT-scan (cardiac)	37 (26.6)	20 (23.5%)	16 (35.6%)	0.155	7 (2–12)
MRI (total)	31 (22.3)	18 (21.2%)	10 (22.2%)	0.665	2 (−1–6)
FDG PET-CT scan	63 (45.3)	26 (30.6%)	27 (60.0%)	<0.001	6 (3–10)

Values expressed as median (interquartile range)

^a^Cardiac-device related IE (CDRIE) cases are also included in the total group

### Surgery

**Fig. 1:**
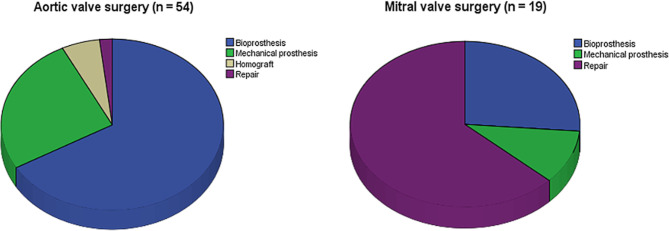
Types of surgical intervention in aortic and mitral valve

A total of 70 patients (50.4%) underwent surgical intervention, i.e. valve surgery or lead removal (Tab. 1). Median time to surgery was 13 days (6–27) after suspected IE diagnosis. Urgent surgery, defined as surgery within 7 days, was performed in 21 patients (15.1%). Early surgery, defined as surgery within 14 days, was performed in 37 patients (26.6%). The most frequently performed procedures were aortic valve surgery (54 patients; 38.8%) and mitral valve surgery (19 patients; 13.7%). Bioprosthetic aortic valve replacement was performed in 36 of the 54 aortic valve procedures (66.7%, Fig. 1). Mitral valve repair was performed in 12 of the 19 mitral valve procedures (63.2%).

### Main outcomes

In-hospital all-cause mortality was 14.4% (median time between suspected IE diagnosis and in-hospital mortality was 30 days, IQR: 14–48) and one-year mortality was 21.6% (see Tab. 3). In-hospital mortality did not differ significantly between surgically and conservatively treated patients (12.9% vs. 15.9%, *P* = 0.391). The incidence of embolic events under treatment was 16.5%. Embolic events were more prevalent in PVE compared to NVE (24.4% vs. 10.6%, *P* = 0.044). Congestive heart failure or cardiogenic shock occurred in 15.1% of the patients, significantly more in NVE patients compared to PVE (20.0% vs. 4.4%, *P* = 0.018). Three patients with cardiogenic shock died in hospital. Of the 23 patients with embolic events during treatment, 7 patients had an indication for surgery but did not receive surgery (because of surgical risk or neurologic complication). Of these 7 patients that were treated conservatively, 5 patients died in-hospital.

**Table 3:** Adverse events under treatment and mortality

	Total *N* (%)	NVE	PVE	*P*-value
Embolic events	23 (16.5)	9 (10.6)	11 (24.4)	0.044
Congestive heart failure or cardiogenic shock	21 (15.1)	17 (20)	2 (4.4)	0.018
Thirty day mortality	11 (7.9)	8 (9.4)	2 (4.4)	0.492
In-hospital mortality	20 (14.4)	11 (12.9)	8 (17.8)	0.449
1‑year mortality	30 (21.6)	17 (20.0)	11 (25.0)	0.509

### Prognostic factors

Univariate analysis was performed to assess association between individual variables and clinical outcome (Suppl. Table 1). The mean age of patients who deceased in-hospital was 72 years, whereas the mean age of the patients who survived was 63 years (*P* = 0.019, 95% CI: 1.52–16.35). Age was associated with in-hospital mortality with an odds ratio of 1.050 per life year. Using multivariate analysis, embolic events during treatment was identified as an independent prognostic factor for in-hospital mortality (OR = 5.551, 95% CI: 1.862–16.547, *P* = 0.002). SA endocarditis was also found to be an independent risk factor (OR = 4.205, 95% CI: 1.468–12.043, *P* = 0.007).

PVE, timing to imaging, surgery and timing to surgery were not associated with increased mortality. Female sex showed a trend towards significant association with in-hospital mortality (*P* = 0.062). Female patients were more likely to have SA endocarditis compared to men (OR = 2.554, 95% CI: 1.121–5.861, *P* = 0.029) and tended to develop embolic complications during treatment more often than men (*P* = 0.068), both which are associated with higher in-hospital mortality.

## Discussion

Since the analysis of bacterial endocarditis by Van der Meer et al. in 1992 this is the first prospective study examining the current mortality of IE in the Netherlands [12]. We observed a relatively low in-hospital mortality of 14.4% and a one-year mortality of 21.6%. In a recent study in a single center without cardiac surgery facilities in the Netherlands, an in-hospital mortality of 18% was reported [13], while other studies reported a one-year mortality around 30% [14, 15]. The group patients that were included in our study were patients in tertiary hospitals with cardiac surgery facilities. This difference in patient population might account for differences in mortality.

Furthermore, reported complications of IE include embolization and heart failure due to valve destruction. A higher incidence of embolic events was observed in PVE patients compared to NVE. It must be noted however, that ^18^F‑FDG PET/CT imaging was significantly more often performed in PVE than in NVE, which may have resulted in more (silent) embolus detection. Conversely, we found more congestive heart failure and cardiogenic shock in NVE. Patients with NVE referred to tertiary centers are more likely to suffer from progressive IE, valve destruction and consecutive heart failure. Because previous studies showed worse prognosis of PVE [16, 17], physicians may treat patients with prosthetic valves and signs of infection more cautiously resulting in early referral and adequate therapy in a timely fashion.

The results of this study implicate a rapid use of imaging modalities once IE is suspected. TTE is usually performed on the same day of cardiologic consultation and TEE follows within a week with a median duration of three days. Although we did not find a significant association between timing of imaging and mortality, prompt timing of imaging is crucial for early decision-making. Another finding with regard to imaging is the extensive use of ^18^F‑FDG PET/CT scan. Knowing that ^18^F‑FDG PET/CT scan has proven its added value in diagnosing PVE and CDRIE as well as in detecting embolic events, further use of this imaging modality may aid in correct diagnosis of IE [8]. Moreover, Swart et al. suggested early use of ^18^F‑FDG PET/CT can increase diagnostic performance in PVE [18]. With a median of six days after IE suspicion, ^18^F‑FDG PET/CT is also rapidly and consistently used.

Interestingly, valve surgery has been carried out in half of the patients referred to cardiac surgery centers. Improved identification of patients eligible for surgery may have contributed to this number. Early surgery has been shown to decrease the composite endpoint of death from any cause and embolic events [19]. In our study, a large distribution is seen in timing of surgery, varying from 1 to 71 days with a median of 13 days after suspicion of IE. Of the patients who underwent surgery, more than half received early surgery (<14 days). In patients with aortic valve endocarditis who went for surgery, bioprosthetic aortic valves were the most frequently used followed by mechanical valves. In patients with infected mitral valves, mitral valve repair was the most commonly performed procedure, which may be related to less valve destruction at time of presentation and ease of surgery. Surgery on patients despite embolic events when indication for surgery exists (i.e. heart failure, uncontrolled infection, abscess or persistent high embolic risk) can be done with low neurological risk, provided cerebral hemorrhage has been excluded on cranial CT [20]. The threshold for surgery in these patients appears high, as one third of the patients with embolic events and indication for surgery have been treated conservatively, mostly because of neurologic complication. The outcome of the non-surgically treated patients with embolic events and indication for surgery is poor, 72% died in hospital.

An Endocarditis Team has been reported to reduce mortality in IE patients, although these results stem from historical control data [21, 22]. In the most recent update of the guidelines for the management of patients with endocarditis, the constitution of an Endocarditis team is recommended as a crucial part in the improvement of care for patients with IE. In five of the seven participating centers in the Netherlands, patients were discussed in an Endocarditis Team. In the other two hospitals patients were discussed in a heart team with consultation of an infectiologist and a medical microbiologist. This multidisciplinary approach may have positively influenced patient outcome.

Looking at the epidemiological characteristics, we see a relatively high proportion of PVE (33.1%). An in-hospital mortality of 22.8% has been reported in PVE [23]. We did not observe an association between PVE and in-hospital or one-year mortality. SA endocarditis is known to increase the risk of in-hospital mortality [24]. Its frequency has risen worldwide over the past decades up to 30%, pre-dominantly driven by SA endocarditis increase in North America (>50%). In Europe current frequency of SA endocarditis is between 20–30% with no clear alteration in the last two decades [25, 26]. In the present study, SA endocarditis comprised 21.6% of the IE population in the Netherlands.

Other baseline characteristics like median age, male to female ratio and percentage positive blood cultures are comparable with previous reports (Suppl. Table 2).

As pointed out earlier, limitations of this registry include the possible selection bias. We identified only those patients referred to a larger hospital with a cardiac surgery department, for evaluation of IE. Patients in non-cardiac surgery hospitals not needed to be transferred were not included in the present study. This might have influenced the patient characteristics and therefore the effect on mortality can be ambiguous; patients with uncomplicated IE with a small vegetation and no indication for surgery are not included and therefore real mortality may be even lower. On the other hand, patients not suitable for surgery because of age, comorbidity or complications and possibly a worse predicted outcome may not have been referred. Including these patients would imply a higher mortality. Furthermore, patients who were critically ill (e.g. clinical unstable patients or treated with a ventilator) at admission and who were not able to give informed consent were also not included. Lastly, due to the limited number of hospitals that participated in the EURO-ENDO study and the varying period of inclusion, we could not comment on the incidence of IE.

Recently the EURO-ENDO registry has been completed and prospective data on IE profile and outcome in different countries are now available [26]. Compared to the European cohort similar results are seen with regard to age, prosthetic valve endocarditis and surgery rate. Differences are observed regarding imaging; TOE and ^18^F‑FDG PET/CT are more frequently used in the Netherlands. Moreover, the in-hospital mortality of IE in the Dutch cohort is slightly lower than the mortality in the overall EURO-ENDO cohort (14.4% vs. 17.1%).

## Conclusion

A relatively low in-hospital and one-year mortality was observed in patients with IE in the Netherlands, despite the large group of PVE and SA endocarditis. Multiple changes in recent years could be the cause; the multidisciplinary management by an Endocarditis Team, the early and extensive use of imaging and the relatively high rate of (early) surgery. However, while the low encountered mortality may indicate a leap in the good direction since the last decades of IE management, we must be careful in drawing conclusions, as some results may be affected by patient selection bias. Further research with inclusion of patients in non-cardiac surgery hospitals is necessary to confirm our findings.

## Caption Electronic Supplementary Material

Suppl. Table 1 Distribution of causative organism

Suppl. Table 2 Prognostic factors for in-hospital mortality

Suppl. Table 3 Changing IE profile in The Netherlands

List of the EURO-ENDO Investigators Group and of the EURO-ENDO National Coordinators
