# Supplementary material for: Infective endocarditis in the Netherlands: current epidemiological profile and mortality: An analysis based on partial ESC EORP collected data
Source: Neth Heart J. 2020 Jun 5;28(10):526–36. doi: 10.1007/s12471-020-01431-z (PMC7494701; doi:10.1007/s12471-020-01431-z)
Supplement: Supplementary file 1 — Suppl. Table 1 Distribution of causative organism [file 12471_2020_1431_MOESM1_ESM.docx]

| **Suppl. Table 1 Distribution of causative organism** | | | | |
| --- | --- | --- | --- | --- |
| **Microorganism** | | **Native valve endocarditis**  **%** | **Prosthetic valve endocarditis**  **%** | ***P*-value** |
| Staphylococcus aureus | | 23.5 | 19.5 | 0.66 |
| Streptococci | | 48.0 | 31.7 | 0.09 |
| Enterococci | | 8.2 | 19.5 | 0.08 |
| HACEK | | 1.0 | 0.0 | 1.00 |
| Coagulase negative Staphylococcus | | 2.0 | 14.6 | < 0.01 |
| Other | | 48.0 | 26.8 | 0.02 |

*HACEK* Haemophilus species, Aggregatibacter species, Cardiobacterium hominis, Eikenella corrodens and Kingella species
