# Supplementary material for: Infective endocarditis in the Netherlands: current epidemiological profile and mortality: An analysis based on partial ESC EORP collected data
Source: Neth Heart J. 2020 Jun 5;28(10):526–36. doi: 10.1007/s12471-020-01431-z (PMC7494701; doi:10.1007/s12471-020-01431-z)
Supplement: Supplementary file 2 — Suppl. Table 2 Prognostic factors for in-hospital mortality [file 12471_2020_1431_MOESM2_ESM.docx]

| **Table 5 Prognostic factors for in-hospital mortality** | |  | |
| --- | --- | --- | --- |
| *Univariate analysis* | **Odds ratio (95% CI)** | | ***P*-value** |
| Age^a^ | 1.054 (1.007-1.103) | | 0.023 |
| Staphylococcus aureus | 4.667 ( 1.725-12.625) | | 0.002 |
| Embolic events^b^ | 6.136 (2.163-17.406) | | 0.001 |
| *Multivariate analysis* |  | |  |
| Staphylococcus aureus | 4.205 (1.468-12.043) | | 0.007 |
| Embolic events^b^ | 5.551 (1.862-16.547) | | 0.002 |

*^a^ Odds ratio per life year.*

*^b^ Embolic events during hospital stay, under treatment.*

*CI* confidence interval
