# Supplementary material for: Infective endocarditis in the Netherlands: current epidemiological profile and mortality: An analysis based on partial ESC EORP collected data
Source: Neth Heart J. 2020 Jun 5;28(10):526–36. doi: 10.1007/s12471-020-01431-z (PMC7494701; doi:10.1007/s12471-020-01431-z)
Supplement: Supplementary file 3 — Suppl. Table 3 Changing IE profile in The Netherlands [file 12471_2020_1431_MOESM3_ESM.docx]

| **Suppl. Table 3 Changing IE profile in the Netherlands** | | | |  |  |  |
| --- | --- | --- | --- | --- | --- | --- |
|  | Van der Meer et al. | Van den Brink et al. | Krul et al. | | | El Kadi et al. |
| **Study type** | Multi-centre prospective observational study  (*n*=439 ) | Nationwide retrospective trend study (*n*=5213)  Sample study (*n*=216) | Single-centre retrospective observational study (*n*=89) | | | Multi-centre prospective observational study (*n*=139) |
| **Study period** | 1986-1988 | 2005-2011 | 2008-2013 | | | 2016-2019 |
| **Age** | 52 | 67.5 | 68 | | | 63.9 |
| **Positive culture (%)** | 94.5 | 90.7 | 92.1 | | | 91.4 |
| **S. aureus (%)** | 21.6 | 30.1 | 24.4 | | | 22.3 |
| **PVE (%)** | 20.3 | 30.1 | 21.3 | | | 32.4 |
| **CDRIE (%)** | N/A | 7.9 | 7.9 | | | 7.2 |
| **Surgery (%)** | N/A | 38.9 | 41.5 | | | 50.4 |
| **Mortality (%)** | 19.7  (in-hospital) | 36.1  (FU 4.2 years) | 18  (in-hospital) | | | 14.4  (in-hospital) |

*IE* infective endocarditis, *PVE* prosthetic valve endocarditis, *CDRIE* cardiac disease related infective endocarditis
