# Supplementary material for: Infective endocarditis in the Netherlands: current epidemiological profile and mortality: An analysis based on partial ESC EORP collected data
Source: Neth Heart J. 2020 Jun 5;28(10):526–36. doi: 10.1007/s12471-020-01431-z (PMC7494701; doi:10.1007/s12471-020-01431-z)
Supplement: Supplementary file 4 — List of the EURO-ENDO Investigators Group and of the EURO-ENDO National Coordinators [file 12471_2020_1431_MOESM4_ESM.docx]

**European Infective Endocarditis registry (EURO-ENDO) - Appendix1**

**EORP Oversight Committee**

Chris P Gale, GB (Chair); Branko Beleslin, RS; Andrzej Budaj, PL; Ovidiu Chioncel, RO; Nikolaos Dagres, DE; Nicolas Danchin, FR; Jonathan Emberson, GB; David Erlinge, SE; Michael Glikson, IL; Alastair Gray, GB; Meral Kayikcioglu, TR; Aldo P. Maggioni, IT; Vivien Klaudia Nagy, HU; Aleksandr Nedoshivin, RU; Anna-Sonia Petronio, IT; Jolien Roos-Hesselink, NL; Lars Wallentin, SE; Uwe Zeymer, DE

**Executive Committee**

Gilbert Habib, FR (Chair); Patrizio Lancellotti, BE (Chair); Bernard Cosyns, BE; Erwan Donal, FR; Paola Erba, IT; Gilbert Habib, FR; Bernard Iung, FR; Aldo P. Maggioni, IT; Bogdan A. Popescu, RO; Bernard Prendergast, GB; Pilar Tornos, ES

**National Coordinators**

Nora Nabila Ali Tatar-Chentir, DZ; Mouaz Al-Mallah, SA; Meriam Astrom Aneq, SE; George Athanassopoulos, GR; Luigi Paolo Badano, IT; Soraya Benyoussef, TN; Erick Calderon Aranda, MX; Nuno Miguel Cardim, PT; Kwan-Leung Chan, CA; Bernard Cosyns, BE; Ines Cruz, PT; Thor Edvardsen, NO; Georg Goliasch, AT; Gilbert Habib, FR; Andreas Hagendorff, DE; Krasimira Hristova, BG; Bernard Iung, FR; Otto Kamp, NL; Duk-Hyun Kang, KR; William Kong, SG; Simon Matskeplishvili, RU; Marwa Meshaal, EG; Maja Mirocevic, ME; Aleksandar N. Neskovic, RS; Michal Pazdernik, CZ; Edyta Plonska-Gosciniak, PL; Bogdan A. Popescu, RO; Bernard Prendergast, GB; Maha Raissouni, MA; Ricardo Ronderos, AR; Leyla Elif Sade, TR; Anita Sadeghpour, IR; Antonia Sambola, ES; Shantanu Sengupta, IN; Jadranka Separovic-Hanzevacki, HR; Masaaki Takeuchi, JP; Edwin Tucay, PH; Ana Clara Tude Rodrigues, BR; Albert Varga, HU; Jolanta Vaskelyte, LT; Kentaro Yamagata, MT; Kyriakos Yiangou, CY; Hosam Zaky, AE

**Investigators**

**Argentina:** *Buenos Aires:* R. Ronderos, G. Avegliano, P. Fernandez Oses, E. Filipini, I. Granada, A. Iribarren, M. Mahia, F. Nacinovich, S. Ressi, *Corrientes:* R. Obregon, M. Bangher, J. Dho, *La Plata:* L. Cartasegna, M.L. Plastino, V. Novas, C. Shigel, *Florencio Varela:* G. Reyes, M. De Santos, N. Gastaldello, M. Granillo Fernandez, M. Potito, G. Streitenberger, P. Velazco, *Buenos Aires:* J.H. Casabé, C. Cortes, E. Guevara, F. Salmo, M. Seijo; **Austria:** *Vienna:* F. Weidinger, M. Heger, R. Brooks, C. Stöllberger, C-Y. Ho, L. Perschy, L. Puskas, *Vienna:* G. Goliasch, C. Binder, R. Rosenhek, M. Schneider, M-P. Winter; **Belgium:** *Liege:* E. Hoffer, M. Melissopoulou, E. Lecoq, D. Legrand, S. Jacquet, M. Massoz, *Liege:* P. Lancellotti, L. Pierard, R. Dulgheru, S. Marchetta, C. D´Emal, C. Oury, *Jette:* B. Cosyns, S. Droogmans, D. Kerkhove, A. Motoc, D. Plein, B. Roosens, L. Soens, C. Weytjens, I. Lemoine, *Edegem:* I. Rodrigus, B. Paelinck, B. Amsel, *Brussels:* P. Unger, D. Konopnicki, *Brussels:* C. Beauloye, A. Pasquet, S. Pierard, D. Vancraeynest, J.L. Vanoverschelde, F. Sinnaeve; **Brazil:** *Sao Paulo:* J.L. Andrade, A.C. Tude Rodrigues, K. Staszko, *Porto Alegre:* R. Dos Santos Monteiro, M.H. Miglioranza, D.L. Shuha, *Rio de Janeiro:* M. Alcantara, V. Cravo, L. Fazzio, A. Felix, M. Iso, C. Musa, A.P. Siciliano, *Marilia:* F. Villaca Filho, J. Braga, A. Rodrigues, R. Silva, F. Vilela, D. Rodrigues, L. Silva, *Sao Paulo:* S. Morhy, C. Fischer, R. Silva, M. Vieira, T. Afonso, *Fortaleza:* J. Abreu, S.N. Falcao, *Sao Paulo:* V. Moises, A. Gouvea, G. João, F. Mancuso, C. Silva, A.C. Souza, *Sao Paulo:* C.S. Abboud, R. Bellio de Mattos Barretto, A. Ramos, R. Arnoni, J.E. Assef, D.J. Della Togna, D. Le Bihan, L. Miglioli, A.P. Romero Oliveira, R. Tadeu Magro Kroll, D. Cortez, *Belo Horizonte:* C.L. Gelape, M.d.C. Peirira Nunes, T.C. De Abreu Ferrari; **Canada:** *Ottawa:* K-L. Chan, K. Hay, *Montreal:* V. Le, M. Page, F. Poulin, C. Sauve, K. Serri, C. Mercure, *Quebec:* J. Beaudoin, P. Pibarot, *Montreal:* I. Sebag, L. Rudski, G. Ricafort; **Croatia:** *Zagreb:* B. Barsic, V. Krajinovic, M. Vargovic, *Zagreb:* J. Separovic-Hanzevacki, D. Lovric, V. Reskovic-Luksic, *Zagreb:* J. Vincelj, S. Jaksic Jurinjak; **Cyprus:** *Nicosia:* V. Yiannikourides, M. Ioannides, C. Kyriakou, C. Pofaides, V. Masoura, K. Yiangou; **Czech Republic:** *Ostrava-Poruba:* J. Pudich, *Prague:* A. Linhart, M. Siranec, J. Marek, *Prague:* K. Blechova, M. Kamenik, *Prague:* M. Pazdernik, *Hradec Kralove:* R. Pelouch, *Zlin:* Z. Coufal, M. Mikulica, M. Griva, E. Jancova, M. Mikulcova, *Olomouc:* M. Taborsky, J. Precek, M. Jecmenova, J. Latal, *Liberec:* J. Widimsky, *Prague:* T. Butta, S. Machacek, *Pilsen:* R. Vancata, *Brno:* J. Spinar, M. Holicka; **Ecuador:** *Guayaquil:* F. Pow Chon Long, N. Anzules, A. Bajana Carpio, G. Largacha, E. Penaherrera, D. Moreira; **Egypt:** *Mansoura:* E. Mahfouz, E. Elsafty, A. Soliman, Y. Zayed, J. Aboulenein, *Alexandria:* M. Abdel-Hay, A. Almaghraby, M. Abdelnaby, M. Ahmed, B. Hammad, Y. Saleh, H. Zahran, O. Elgebaly, *Zagazig:* A. Saad, M. Ali, *Alexandria:* A. Zeid, R. El Sharkawy, *Cairo:* M. Meshaal, A. Al Kholy, R. Doss, D. Osama, H. Rizk, A. Elmogy, M. Mishriky; **France:** *Kremlin-Bicêtre:* P. Assayag, S. El Hatimi, *Saint-Etienne:* E. Botelho-Nevers, S. Campisi, J-F. Fuzellier, A. Gagneux-Brunon, R. Pierrard, C. Tulane, M. Detoc, T. Mehalla, *Nantes:* D. Boutoille, O. Al Habash, N. Asseray-Madani, C. Biron, J. Brochard, J. Caillon, C. Cueff, T. Le Tourneau, A.S. Lecompte, R. Lecomte, M. Lefebvre, M.M. Magali Michel, S. Pattier, S. Delarue, M. Le Bras, J. Orain, *Limoges:* J-F. Faucher, V. Aboyans, A. Beeharry, H. Durox, M. Lacoste, J. Magne, D. Mohty, A. David, V. Pradel, *Thonon-les-Bains:* V. Sierra, A. Neykova, B. Bettayeb, S. Elkentaoui, B. Tzvetkov, G. Landry, *Reims:* C. Strady, K. Ainine, S. Baumard, C. Brasselet, C. Tassigny, V. Valente-Pires, M. Lefranc, *Pointe-à-Pitre:* B. Hoen, B. Lefevre, E. Curlier, C. Callier, N. Fourcade, *Brest:* Y. Jobic, S. Ansard, R. Le Berre, P. Le Roux, F. Le Ven, M-C. Pouliquen, G. Prat, *Rouen:* F. Bouchart, A. Savoure, C. Alarcon, C. Chapuzet, I. Gueit, *Amiens:* C. Tribouilloy, Y. Bohbot, F. Peugnet, M. Gun, *Paris:* B. Iung, X. Duval, X. Lescure, E. Ilic-Habensus, *Nancy:* N. Sadoul, C. Selton-Suty, F. Alla, E. Chevalier, F. Goehringer, O. Huttin, *Poitiers:* R. Garcia, V. Le Marcis, *Rennes:* P. Tattevin, E. Donal, E. Flecher, M. Revest, *Marseille:* G. Habib, *Besançon:* C. Chirouze, K. Bouiller, A-S. Brunel, D. Fournier, L. Hustache-Mathieu, T. Klopfenstein, J. Moreau, *Créteil:* P. Lim, L. Oliver, J. Ternacle, A. Moussafeur, *Dijon:* P. Chavanet, L. Piroth, M. Buisson, S. Mahy, C. Martins, A. Salmon-Rousseau, S. Gohier; **Germany:** *Bad Oeynhausen:* C. Piper, J. Börgermann, D. Guckel, D. Horstkotte, B. Brockmeier, E. Winkelmann, *Leipzig:* A. Hagendorff, D. Grey, *Bonn:* G. Nickenig, R. Schueler, C. Öztürk, E. Stöhr, *Bad Nauheim:* C. Hamm, T. Walther, R. Brandt, A-C. Frühauf, C.T. Hartung, C. Hellner, C. Wild, *Aachen:* M. Becker, S. Hamada, W. Kaestner, *Berlin:* K. Stangl, F. Knebel, G. Baldenhofer, A. Brecht, H. Dreger, C. Isner, F. Pfafflin, M. Stegemann, *Ludwigshafen:* R. Zahn, B. Fraiture, C. Kilkowski, A-K. Karcher, S. Klinger, H. Tolksdorf; **Greece:** *Athens:* D. Tousoulis, C. Aggeli, G. Sarri, S. Sideris, E. Venieri, *Athens:* G. Athanassopoulos, D. Tsiapras, I. Armenis, A. Koutsiari, *Athens:* G. Floros, C. Grassos, S. Dragasis, *Athens:* L. Rallidis, C. Varlamos, *Ioannina:* L. Michalis, K. Naka, A. Bechlioulis, A. Kotsia, L. Lakkas, K. Pappas, *Athens:* C. Papadopoulos, S. Kiokas, A. Lioni, S. Misailidou, *Athens:* J. Barbetseas, M. Bonou, C. Kapelios, I. Tomprou, K. Zerva, *Voula:* A. Manolis, E. Hamodraka, D. Athanasiou, G. Haralambidis, L. Poulimenos, H. Samaras; **Hungary:** *Budapest:* A. Nagy, A. Bartykowszki, E. Gara; **India:** *Nagpur:* S. Sengupta, K. Mungulmare, *Gurgaon:* R. Kasliwal, M. Bansal, A. Bhan, S. Ranjan; **Iran:** *Tehran:* M. Kyavar, M. Maleki, F. Noohi Bezanjani, A. Sadeghpour, A. Alizadehasl, S. Boudagh, A. Ghavidel, P. Moradnejad, H.R. Pasha, B. Ghadrdoost; **Israel:** *Jerusalem:* D. Gilon, J. Strahilevitz, S. Israel, M. Wanounou; **Italy:** *Bari:* C. d'Agostino, P. Colonna, L. De Michele, F. Fumarola, M. Stante, *Florence:* N. Marchionni, V. Scheggi, B. Alterini, S. Del Pace, P. Stefano, C. Sparano, *Padova:* L.P. Badano, D. Muraru, N. Ruozi, R. Tenaglia, *Grosseto:* U. Limbruno, A. Cresti, P. Baratta, M. Solari, *Milan:* C. Giannattasio, A. Moreo, B. De Chiara, B. Lopez Montero, F. Musca, C.A. Orcese, F. Panzeri, C.F. Russo, F. Spano, *Milan:* O. Alfieri, M. De Bonis, E. Agricola, E. Busnardo, S. Carletti, B. Castiglioni, S. Chiappetta, B. Del Forno, D. Ferrara, M. Guffanti, G. Iaci, E. Lapenna, T. Nisi, C. Oltolini, U. Pajoro, R. Pasciuta, M. Ripa, P. Scarpellini, C. Tassan Din, R. Meneghin, D. Schiavi, *Salerno:* F. Piscione, R. Citro, R.M. Benvenga, L. Greco, C. Prota, I. Radano, L. Soriente, M. Bellino, D. Di Vece, *Genoa:* F. Santini, A. Salsano, G.M. Olivieri, *Modena:* F. Turrini, R. Messora, *Modena:* S. Tondi, A. Olaru, V. Agnoletto, L. Grassi, C. Leonardi, S. Sansoni, *Turin:* S. Del Ponte, G.M. Actis Dato, A. De Martino; **Japan:** *Nagoya:* N. Ohte, S. Kikuchi, K. Wakami, *Tsukuba:* K. Aonuma, Y. Seo, T. Ishizu, T. Machino-Ohtsuka, M. Yamamoto, N. Iida, H. Nakajima, *Tenri:* Y. Nakagawa, C. Izumi, M. Amano, M. Miyake, K. Takahashi, *Osaka:* I. Shiojima, Y. Miyasaka, H. Maeba, Y. Suwa, N. Taniguchi, S. Tsujimoto, *Kobe:* T. Kitai, M. Ota, *Sapporo:* S. Yuda, S. Sasaki, *Tokyo:* N. Hagiwara, K. Yamazaki, K. Ashihara, K. Arai, C. Saitou, S. Saitou, G. Suzuki, *Miyazaki:* Y. Shibata, N. Watanabe, S. Nishino, K. Ashikaga, N. Kuriyama, *Tokyo:* K. Mahara, K. Abe, H. Fujimaki, T. Okubo, H. Shitan, S. Takanashi, M. Terada, H. Yamamoto, *Tokushima:* M. Sata, H. Yamada, K. Kusunose, Y. Saijo, H. Seno, O. Yuichiro, *Suita:* Y. Sakata, H. Mizuno, S. Nakatani, T. Onishi, K. Sengoku, F. Sera; **Korea, Republic Of:** *Seoul:* S.W. Park, K. Eun Kyoung, L. Ga Yeon, J-w. Hwang, C. Jin-Oh, S-J. Park, L. Sang-Chol, C. Sung-A, S.Y. Jang, *Seoul:* D-H. Kang, R. Heo, S. Lee, J-M. Song, E. Jung; **Lithuania:** *Siauliai:* J. Plisiene, A. Dambrauskaite, G. Gruodyte, *Kaunas:* R. Jonkaitiene, J. Vaskelyte, V. Mizariene, J. Atkocaityte, R. Zvirblyte; **Luxembourg:** *Luxembourg:* R. Sow, A. Codreanu, E.C.L. De la Vega, C. Michaux, T. Staub, L. Jacobs-Orazi; **Malta:** *Msida:* C. Mallia Azzopardi, R.G. Xuereb, T. Piscopo, D. Borg, R. Casha, J. Farrugia, M. Fenech, E. Pllaha, C. Vella, K. Yamagata; **Moldova, Republic Of:** *Chisinau:* L. Grib, E. Raevschi, A. Grejdieru, G. Balan, I. Cardaniuc, L. Cardaniuc, V. Corcea, A. Feodorovici, V. Gaina, L. Girbu, P. Jimbei, D. Kravcenco, E. Panfile, E. Prisacari, E. Samohvalov, S. Samohvalov, N. Sceglova, I. Benesco, V. Marian, N. Sumarga; **Montenegro:** *Podgorica:* M. Mirocevic, B. Bozovic, N. Bulatovic, P. Lakovic, L. Music; **Netherlands:** *Rotterdam:* J. Roos-Hesselink, R. Budde, T. Gamela, A. Wahadat, *Amsterdam:* O. Kamp, T. Meijers, *Groningen:* J.P. Van Melle, V.M. Deursen, *Maastricht:* H. Crijns, S. Bekkers, E. Cheriex, M. Gilbers, B. Kietselaer, C. Knackstedt, R. Lorusso, S. Schalla, S. Streukens, *Utrecht:* S. Chamuleau, M-J. Cramer, A. Teske, T. Van der Spoel, A. Wind, O. Liesbek, J. Lokhorst, H. Van Heusden, *The Hague:* W. Tanis, I. Van der Bilt, J. Vriend, H. De Lange-van Bruggen, E. Karijodikoro, *Amsterdam:* R. Riezebos, E. van Dongen, J. Schoep, V. Stolk; **New Caledonia:** *Noumea:* O. Axler, F. Baumann, S. Lebras; **Norway:** *Oslo:* T. Edvardsen, J.T. Offstad, J.O. Beitnes, T. Helle-Valle, H. Skulstad, R. Skardal; **Pakistan:** *Karachi:* N. Qamar, S. Furnaz, B. Ahmed, M.H. Butt, M.F. Khanzada, T. Saghir, A. Wahid; **Poland:** *Warsaw:* T. Hryniewiecki, P. Szymanski, K. Marzec, M. Misztal-Ogonowska, *Wroclaw:* W. Kosmala, M. Przewlocka-Kosmala, A. Rojek, K. Woznicka, J. Zachwyc, *Bialystok:* A. Lisowska, M. Kaminska, *Lodz:* J. Kasprzak, E. Kowalczyk, D.F. Strzecka, P. Wejner-Mik; **Portugal:** *Carnaxide:* M. Trabulo, P. Freitas, S. Ranchordas, G. Rodrigues, *Guilhufe:* P. Pinto, C. Queiros, J. Azevedo, L. Marques, D. Seabra, *Lisbon:* L. Branco, J. Abreu, M. Cruz, A. Galrinho, R. Moreira, P. Rio, A.T. Timoteo, M. Selas, *Lisbon:* N.M. Cardim, V. Carmelo, B. Duque Neves, *Almada:* H. Pereira, I. Cruz, A. Guerra, A. Marques, I. Pintassilgo; **Romania:** *Timisoara:* M.C. Tomescu, N-M. Trofenciuc, M. Andor, A. Bordejevic, H.S. Branea, F. Caruntu, L. Cirin, I.M. Citu, C.A. Cotoraci, D. Darabantiu, R. Farcas, I. Marincu, A. Mavrea, M.F. Onel, T. Parvanescu, D. Pop, A.L. Pop-Moldovan, M.I. Puticiu, L.A. Velcean, *Timisoara:* A. Ionac, D. Cozma, C. Mornos, F. Goanta, I. Popescu, *Cluj-Napoca:* R. Beyer, R. Mada, R. Rancea, H. Rosianu, R. Tomoaia, C. Stanescu; **Russian Federation:** *Moscow:* Z. Kobalava, J. Karaulova, E. Kotova, A. Milto, A. Pisaryuk, N. Povalyaev, M. Sorokina; **Saudi Arabia:** *Jeddah:* J. Alrahimi, A. Elshiekh, *Riyadh:* A. Jamiel, A. Ahmed, M. Al-Mallah, N. Attia; **Serbia:** *Belgrade:* B. Putnikovic, A. Neskovic, A. Dimic, *Belgrade:* B. Ivanovic, S. Matic, D. Trifunovic, J. Petrovic, *Belgrade:* D. Kosevic, P. Dabic, P. Milojevic, I. Petrovic, I. Stojanovic, *Sremska Kamenica:* I. Srdanovic, M. Kovacevic, A. Redzek, M. Stefanovic, S. Susak, L. Velicki, A. Vulin; **Singapore:** *Singapore:* T.C. Yeo, W. KF Kong, K.K. Poh; **Spain:** *Madrid:* I. Vilacosta, M. Abd El- Nasser, C. Ferrera, C. Olmos, *Vigo - Pontevedra:* F. Calvo Iglesias, E. Blanco-Gonzalez, M. Bravo Amaro, A.N. Germinas, E. Lopez-Rodriguez, J. Lugo Adan, P. Pazos-Lopez, M. Pereira Loureiro, M.T. Perez, S. Raposeiras-Roubin, S. Rasheed Yas, M-M. Suarez-Varela, F. Vasallo Vidal, *Barcelona:* D. Garcia-Dorado, A. Sambola, N. Fernandez-Hidalgo, T. Gonzalez-Alujas, J. Lozano, O. Maisterra, N. Pizzi, R. Rios, P. Tornos, *Badalona:* A. Bayes-Genis, L. Pedro Botet, N. Vallejo, E. Berastegui, C. Llibre, L. Mateu, R. Nunez, D. Quesada, *Girona:* D. Bosch Portell, J. Aboal Vinas, X. Albert Bertran, R. Brugada Tarradellas, P. Loma-Osorio Ricon, C. Tiron de Llano, *Valencia:* M.A. Arnau, A. Bel, M. Blanes, A. Osa, *Cordoba:* M. Anguita, F. Carrasco, J. Castillo, *Madrid:* J.L. Zamorano, J.L. Moya Mur, M. Alvaro, C. Fernandez-Golfin, J.M. Monteagudo, E. Navas Elorza, *Santander:* M.C. Farinas Alvarez, J. Aguero Balbin, C. Arminanzas, F. Arnaiz de las Revillas, A. Arnaiz Garcia, M. Cobo Belaustegui, M. Fernandez Sampedro, M. Gutierrez Cuadra, J.F. Gutierrez-Diez, J. Zarauza, L. Garcia Cuello, C. Gonzalez Rico, *Barakaldo:* R. Rodriguez-Alvarez, J. Goikoetxea, M. Montejo, *Barcelona:* J. Miro, M. Almela, J. Ambrosioni, C. Falces, D. Fuster, C. Garcia-de-la-Maria, M. Hernandez-Meneses, J. Llopis, F. Marco, A. Moreno, E. Quintana, E. Sandoval, A. Tellez, J.M. Tolosana, B. Vidal, I. Ruiz-Zamora, *Tarragona:* A. Bardaji Ruiz, E. Sanz Girgas, G. Garcia-Pardo, M. Guillen Marzo, A. Rodriguez Oviedo, A. Villares Jimenez; **Tunisia:** *Sfax:* L. Abid, R. Hammami, S. Kammoun, *Tunis:* M.S. Mourali, F. Mghaieth Zghal, M. Ben Hlima, S. Boudiche, S. Ouali, *La Marsa:* L. Zakhama, S. Antit, I. Slama; **Turkey:** *Samsun:* O. Gulel, M. Sahin, *Ankara:* L.E. Sade, E. Karacaglar, *Istanbul:* S. Kucukoglu, O. Cetinarslan, U.S. Yasar, *Ankara:* U. Canpolat, *Istanbul:* B. Mutlu, H. Atas, R. Dervishova, C. Ileri; **United Arab Emirates:** *Dubai:* H. Zaky, J. Alhashmi, F. Baslib, J. Tahir, P. Zarger; **United Kingdom:** *London:* S. Woldman, L. Menezes, C. Primus, R. Uppal, I. Bvekerwa, *Swindon:* B. Chandrasekaran, A. Kopanska, *London:* B. Prendergast, S. Cannata, J. Chambers, J. Hancock, J. Klein, R. Rajani, M.P. Ursi, *London:* R. Dworakowski, A. Fife, J. Breeze, M. Browne-Morgan, M. Gunning, S. Streather; **United States:** *Washington:* F. Asch, M. Zemedkun; **Uzbekistan:** *Tashkent:* B. Alyavi, J. Uzokov
